# Surgical Management for Acute Ischemic Colitis Associated with Decompression Sickness

**DOI:** 10.70352/scrj.cr.24-0185

**Published:** 2025-04-10

**Authors:** Daisaku Kamiimabeppu, Kenji Baba, Masumi Wada, Naoki Kuroshima, Yota Kawasaki, Ken Sasaki, Takaaki Arigami, Ikumi Kitazono, Takao Ohtsuka

**Affiliations:** 1 Department of Gastrointestinal Surgery, Graduate School of Medical and Dental Sciences, Kagoshima University, Kagoshima, Kagoshima, Japan; 2 Department of Pathology, Graduate School of Medical and Dental Sciences, Kagoshima University, Kagoshima, Kagoshima, Japan

**Keywords:** ischemic colitis, air embolism, decompression sickness, scuba diving, surgical management

## Abstract

**INTRODUCTION:**

Ischemic colitis secondary to decompression sickness (DCS) is rare. Here, we present a case of ischemic colitis resulting in bowel necrosis following DCS.

**CASE PRESENTATION:**

A 63-year-old male, with a history of hyperbaric oxygen (HBO) therapy for DCS 6 years ago, presented with limb and lower abdominal pain after a 55-m dive. The patient was diagnosed with DCS, and HBO therapy was initiated. However, due to worsening lower abdominal pain, contrast-enhanced computed tomography was performed on the second day. Imaging revealed a poorly enhanced segment extending from the rectum to sigmoid colon suggestive of bowel necrosis. Emergency surgery was performed, and the necrotic bowel segments were resected, followed by a descending colostomy. Pathological examination revealed ischemic colitis.

**CONCLUSIONS:**

Ischemic colitis should be considered a differential diagnosis in patients with DCS presenting with abdominal symptoms. Surgical intervention may be required in patients with recurrent DCS, depending on the patient’s condition.

## Abbreviations

CT
: computed tomography
DCS
: decompression sickness
HBO
: hyperbaric oxygen
IC
: ischemic colitis

## INTRODUCTION

Ischemic colitis (IC) is a serious condition characterized by reduced blood flow to the colon.^1)^ It is transient and non-gangrenous in most patients and resolves without sequelae. Some patients develop colonic necrosis and gangrene, which can be life-threatening, making rapid diagnosis and treatment imperative. It most often affects older adults and may be more prevalent in women.^2)^ However, IC can also occur as a complication of decompression sickness (DCS) or barotrauma-related air embolism. DCS is a well-recognized risk in divers and is caused by the formation of gas bubbles in tissues and blood vessels due to rapid changes in pressure.^3)^ The most common manifestations of DCS include pain, numbness, skin rash and paresthesia.^4)^ Gastrointestinal symptoms, which account for 2.8% of all DCS patients, are less common but can be severe and may require surgical intervention when they occur. Herein, we report the surgical management of IC resulting in bowel necrosis following DCS.

## CASE PRESENTATION

**Fig. 1:**
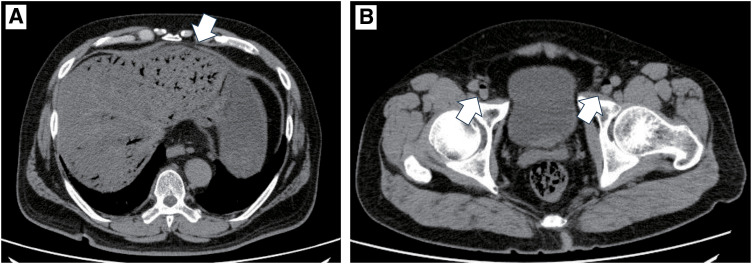
A large amount of intravenous gas was detected in the (**A**) portal vein (white arrow) and (**B**) lateral femoral vein (white arrow). These images were obtained upon initial presentation after the dive.

**Fig. 2:**
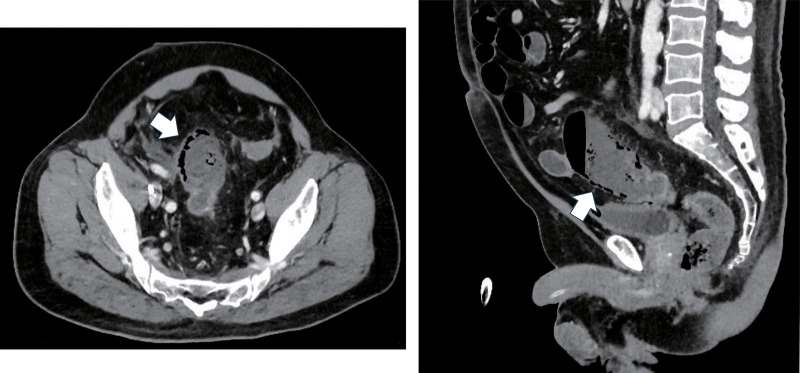
Computed tomography (CT) imaging revealed pneumatosis intestinalis of the sigmoid colon (white arrow). This CT scan was performed on the second day after the dive when symptoms had worsened.

**Fig. 3:**
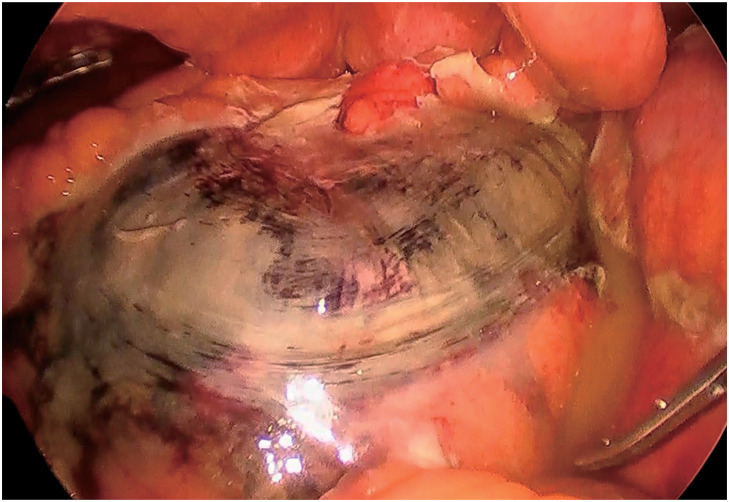
Operative findings showed focal serosal necrosis over the sigmoid colon.

**Fig. 4:**
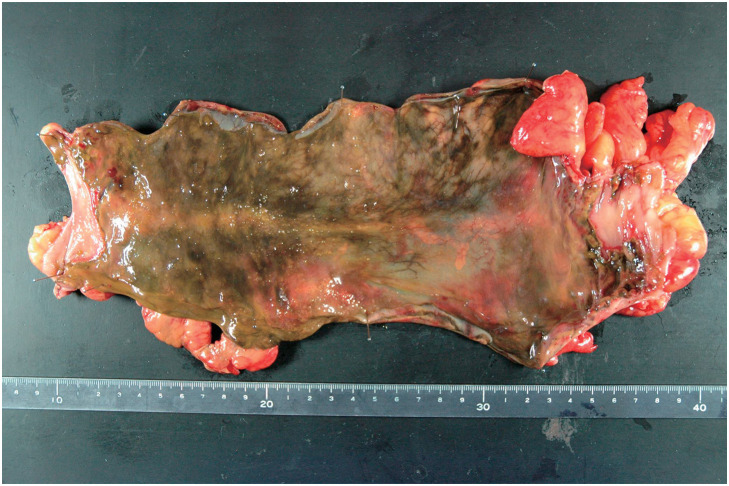
Resected sigmoid colon. Ischemic changes were observed in all layers.

**Fig. 5:**
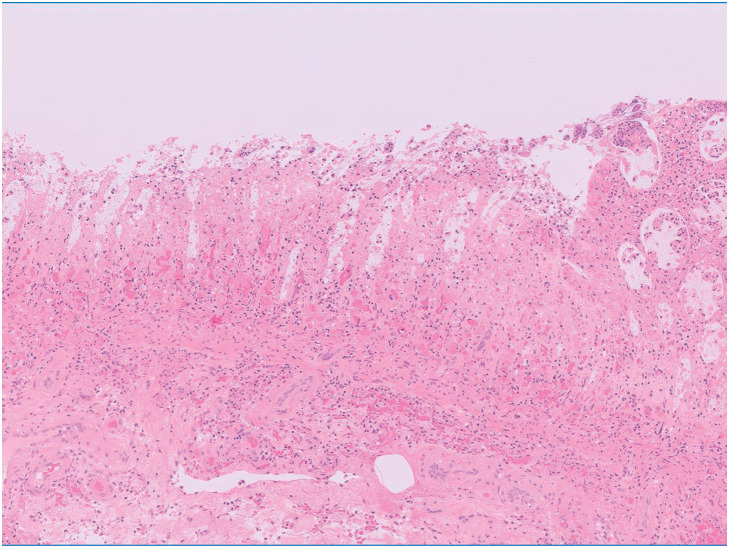
Microscopic findings of the resected colon. Hematoxylin and eosin staining demonstrating transmural ischemic necrosis with significant hemorrhage and inflammatory cell infiltration.

A 63-year-old man, with a history of hypertension and DCS, worked as a scuba instructor. He experienced acute limb and lower abdominal pain immediately after a 55-m dive. The patient was transported to the emergency room. Computed tomography (CT) revealed extensive intravenous gas throughout the body, including in the portal, superior mesenteric, and femoral veins (**Figs. 1A** and **1B**). The patient was diagnosed with DCS and underwent hyperbaric oxygen (HBO) therapy. Two days after admission, the patient complained of worsening lower abdominal pain. On examination, his vital signs were as follows: conscious and alert; body temperature, 36.7°C; blood pressure, 142/76 mmHg; pulse rate, 72 beats per minute; respiratory rate, 22 breaths per minute; and oxygen saturation, 98% on 3 L of oxygen via a nasal cannula. Contrast-enhanced CT revealed poor contrast enhancement from the sigmoid colon to the rectum, suggesting colorectal necrosis (**Fig. 2**). Therefore, emergency surgery was performed. Intraoperative findings revealed focal serosal necrotic changes in the sigmoid colon and rectum (**Fig. 3**). No ischemic changes were observed in the other intestinal segments. Subsequently, a descending colostomy was performed after partial colon resection of approximately 30 cm. Blood flow was confirmed by indocyanine green fluorescence. Pathological examination of the resected colon revealed hemorrhagic bowel necrosis (**Fig. 4**). Microscopic examination revealed transmural ischemic necrosis, with significant hemorrhage and inflammatory cell infiltration (**Fig. 5**). Postoperatively, the patient developed catheter-related infections and fistula formation at the stoma site. However, these complications resolved. The patient was transferred to another hospital 28 days postoperatively.

## DISCUSSION

DCS results from nitrogen bubbles formed in the body owing to rapid pressure changes during decompression.^3)^ The common symptoms include joint pain, fatigue, and skin rashes. Unfortunately, severe DCS can affect the nervous and cardiovascular systems.^4)^ Gastrointestinal symptoms account for approximately 2.8% ^3)^ of all DCS cases. In this case, the patient developed IC leading to intestinal necrosis, which is a rare complication of DCS. The mechanism is unclear, but the formation of bubbles within blood vessels during decompression may obstruct blood flow, causing intestinal ischemia. Additionally, these bubbles may trigger a systemic inflammatory response, potentially exacerbating the ischemic damage.^5)^ Micro-congestive ischemia from mechanical embolisms and endothelial damage induces vascular hyperpermeability and hypercoagulation due to intravascular bubbles,^6)^ suggesting that a combination of these factors can exacerbate ischemic injury.

IC is caused by a reduction in blood flow to a level insufficient to maintain cellular metabolic function, and most often affects older adults.^1,2)^ In general, approximately 15% of patients with colonic ischemia develop a necrotic bowel, the consequences of which can be life-threatening, making rapid diagnosis and treatment imperative. Most cases of colonic ischemia are transient and resolve without sequelae.^7)^ Conservative treatments for IC include fluid resuscitation, bowel rest, and sometimes antibiotics. HBO is the primary treatment for DCS.^8)^ In cases of mild IC associated with DCS, conservative treatment may be sufficient.^9,10)^ However, in our case, the worsening abdominal pain and the finding of poor contrast enhancement on CT were strong indicators of bowel necrosis, necessitating surgical intervention.

We searched the PubMed database for studies published in English, and there are four case reports of IC due to DCS in the literature.^9–12)^ The details of these cases are summarized in **Table 1**. The median (range) patient age was 58 (27–63) years, and four patients were male. Diving depths ranged from 20 to 55 m. Two cases had a history of DCS. Diving deeper than 21 m and a history of DCS are considered as factors that increase the risk of DCS.^13)^ Three cases, including the present case, have been reported to require surgical intervention due to intestinal necrosis. In these cases, the period from onset to surgery was at least 1 day. It is necessary to carefully monitor abdominal symptoms even after hospitalization, and timely intervention with appropriate treatment is important. Moreover, two out of three cases requiring surgery had a history of DCS. Although evidence remains limited, recurrent DCS is generally associated with an increased risk of severe manifestations, necessitating more vigilant monitoring in affected individuals. In patients with a history of DCS, careful observation is essential for timely diagnosis and appropriate management of severe complications, including ischemic colitis. Considering the potential risk, preventive strategies should be considered for recurrent DCS patients. Such preventive measures include optimized dive profiles with controlled ascent rates and adequate pre-dive hydration.^6)^ It is important to acknowledge the limitations of a single case report, as our findings may not be generalizable to all patients with IC following DCS. Further research is needed to elucidate the risk factors, optimal management strategies, and clinical outcomes.

**Table 1:** Previous reports of ischemic colitis due to decompression sickness (DCS)

Case	Author	Publication year	Age	Sex	History of DCS	Diving depths	Extent of resection	Time interval to surgery
1	Goumas et al.^10)^	2008	27	M	No	20 meters	–	–
2	Payor et al.^11)^	2011	53	F	No	22 meters	–	–
3	Choi et al.^9)^	2020	58	M	No	30 meters	Subtotal colon	2 days
4	Toyota et al.^12)^	2020	59	M	Yes	30 meters	T	1 day
5	Our case	–	63	M	Yes	55 meters	S	2 days

## CONCLUSION

Patients with DCS presenting with abdominal symptoms may be at risk of developing IC. Surgical intervention should be considered in severe cases, including DCS recurrence.

## ACKNOWLEDGMENTS

The authors thank Editage (http://www.editage.com) for English-language editing of our manuscript.

## DECLARATIONS

### Funding

This study did not receive any specific grants from funding agencies in the public, commercial, or nonprofit sectors.

### Authors’ contributions

DK drafted the manuscript.

DK, KB, MW, NK, YK, KS, and TA managed the perioperative course and collected data.

IK contributed to the pathological analyses.

TO supervised writing of the manuscript.

All authors have discussed the contents of the manuscript and have read and approved the final version.

### Availability of data and materials

Data availability is not applicable to this study because datasets were not generated or analyzed.

### Ethics approval and consent to participate

Not applicable.

### Consent for publication

Written informed consent was obtained from the patient for the publication of this article and any accompanying images.

### Competing interests

TA received lecture fees from Bristol Myers Squib and Daiichi Sankyo in Japan. The other authors declare that they have no conflicts of interest related to this study.
